# Supplementary material for: Interactions between a subset of substrate side chains and AAA+ motor pore loops determine grip during protein unfolding
Source: eLife. 2019 Jun 28;8:e46808. doi: 10.7554/eLife.46808 (PMC6677533; doi:10.7554/eLife.46808)
Supplement: Figure 2—source data 1. — Values are averages of three biological replicates ± S.D. [file elife-46808-fig2-data1.docx]

**Figure 2—source data 1 – Stimulation of ClpXP ATP hydrolysis by purified substrates**

Values are averages of three biological replicates ± S.D.

| **Substrate** | **ATP hydrolysis (min^-1^ hex^-1^)** | **ATP hydrolyzed  per substrate degraded** |
| --- | --- | --- |
| **ClpXP alone** | 73 ± 4 |  |
| **Gly_12_** | 160 ± 10 |  |
| **GA** | 290 ± 20 | 120 ± 10 |
| **Titin** | 300 ± 20 | 130 ± 10 |
| **Basic** | 240 ± 20 | 220 ± 20 |
| **Acidic** | 320 ± 20 | 120 ± 10 |
| **Tyr1** | 170 ± 10 |  |
| **Tyr2** | 180 ± 10 | 1900 ± 500 |
| **Tyr3** | 190 ± 20 | 260 ± 70 |
| **Tyr4** | 300 ± 20 | 180 ± 10 |
| **Tyr5** | 240 ± 20 | 230 ± 30 |
| **Tyr6** | 280 ± 20 | 3700 ± 800 |
| **Tyr7** | 270 ± 20 |  |
| **Tyr8** | 300 ± 20 |  |
| **Ala4** | 280 ± 20 | 2100 ± 900 |
| **Arg4** | 270 ± 20 | 700 ± 200 |
| **Asn4** | 180 ± 20 |  |
| **Asp4** | 170 ± 10 |  |
| **Cys4** | 400 ± 10 | 2500 ± 600 |
| **Glu4** | 240 ± 20 | 2000 ± 1000 |
| **Gln4** | 270 ± 20 | 700 ± 100 |
| **Ile4** | 290 ± 30 | 200 ± 40 |
| **Leu4** | 300 ± 20 | 230 ± 20 |
| **Lys4** | 240 ± 20 | 800 ± 300 |
| **Met4** | 290 ± 20 | 230 ± 30 |
| **Phe4** | 290 ± 20 | 200 ± 30 |
| **Pro4** | 260 ± 10 |  |
| **Ser4** | 320 ± 10 |  |
| **Thr4** | 230 ± 20 | 2400 ± 600 |
| **Trp4** | 260 ± 20 | 540 ± 50 |
| **Val4** | 300 ± 20 | 180 ± 30 |
| **Ala1** | 160 ± 10 | 800 ± 200 |
| **Ala1+4** | 270 ± 20 | 120 ± 10 |
| **Ala3+4** | 270 ± 20 | 110 ± 10 |
| **Ala4+5** | 240 ± 20 | 140 ± 20 |
| **Ala4+7** | 290 ± 20 | 800 ± 200 |
| **Ala4+9** | 480 ± 40 | 5000 ± 2000 |
| **Tyr1+4** | 520 ± 10 | 350 ± 30 |
| **Tyr2+4** | 480 ± 20 | 230 ± 10 |
| **Tyr3+4** | 400 ± 10 | 330 ± 30 |
| **Tyr4+5** | 440 ± 20 | 320 ± 40 |
| **Tyr4+6** | 520 ± 20 | 210 ± 20 |
| **Tyr4+7** | 410 ± 20 | 420 ± 30 |
| **Tyr4+8** | 510 ± 30 | 250 ± 40 |
| **Tyr1+3** | 380 ± 20 | 270 ± 20 |
| **Tyr2+3** | 410 ± 20 | 510 ± 30 |
| **Tyr3+5** | 240 ± 10 | 210 ± 20 |
| **Tyr3+6** | 310 ± 10 | 420 ± 70 |
| **Tyr3+7** | 300 ± 10 | 270 ± 30 |
| **Tyr3+8** | 380 ± 10 | 390 ± 30 |
| **Val1+4** | 450 ± 10 | 190 ± 10 |
| **Val2+4** | 390 ± 10 | 220 ± 10 |
| **Val3+4** | 390 ± 10 | 250 ± 10 |
| **Val4+5** | 430 ± 10 | 200 ± 10 |
| **Val4+6** | 390 ± 10 | 280 ± 20 |
| **Val4+7** | 420 ± 10 | 320 ± 30 |
| **Val4+8** | 470 ± 10 | 460 ± 30 |
